# Supplementary material for: Development of a computer-aided design software for the quantitative evaluation of aesthetic damage
Source: PLoS One. 2019 Dec 18;14(12):e0226322. doi: 10.1371/journal.pone.0226322 (PMC6919621; doi:10.1371/journal.pone.0226322)
Supplement: S1 Code site — 1. (ZIP) [file pone.0226322.s001.zip › [EESC_jr][Projeto]Relatorio_07_21/reports/templates_zip/gentelella-master/vendors/morris.js/examples/quarters.html]

# Formatting Dates with Quarters

```
/* data stolen from http://howmanyleft.co.uk/vehicle/jaguar_e_type */
var quarter_data = [
  {"period": "2011 Q3", "licensed": 3407, "sorned": 660},
  {"period": "2011 Q2", "licensed": 3351, "sorned": 629},
  {"period": "2011 Q1", "licensed": 3269, "sorned": 618},
  {"period": "2010 Q4", "licensed": 3246, "sorned": 661},
  {"period": "2010 Q3", "licensed": 3257, "sorned": 667},
  {"period": "2010 Q2", "licensed": 3248, "sorned": 627},
  {"period": "2010 Q1", "licensed": 3171, "sorned": 660},
  {"period": "2009 Q4", "licensed": 3171, "sorned": 676},
  {"period": "2009 Q3", "licensed": 3201, "sorned": 656},
  {"period": "2009 Q2", "licensed": 3215, "sorned": 622},
  {"period": "2009 Q1", "licensed": 3148, "sorned": 632},
  {"period": "2008 Q4", "licensed": 3155, "sorned": 681},
  {"period": "2008 Q3", "licensed": 3190, "sorned": 667},
  {"period": "2007 Q4", "licensed": 3226, "sorned": 620},
  {"period": "2006 Q4", "licensed": 3245, "sorned": null},
  {"period": "2005 Q4", "licensed": 3289, "sorned": null},
  {"period": "2004 Q4", "licensed": 3263, "sorned": null},
  {"period": "2003 Q4", "licensed": 3189, "sorned": null},
  {"period": "2002 Q4", "licensed": 3079, "sorned": null},
  {"period": "2001 Q4", "licensed": 3085, "sorned": null},
  {"period": "2000 Q4", "licensed": 3055, "sorned": null},
  {"period": "1999 Q4", "licensed": 3063, "sorned": null},
  {"period": "1998 Q4", "licensed": 2943, "sorned": null},
  {"period": "1997 Q4", "licensed": 2806, "sorned": null},
  {"period": "1996 Q4", "licensed": 2674, "sorned": null},
  {"period": "1995 Q4", "licensed": 1702, "sorned": null},
  {"period": "1994 Q4", "licensed": 1732, "sorned": null}
];
Morris.Line({
  element: 'graph',
  data: quarter_data,
  xkey: 'period',
  ykeys: ['licensed', 'sorned'],
  labels: ['Licensed', 'SORN']
});
```
